# Pharmacist-led responses to headache and insomnia symptoms: A simulated patient study

**DOI:** 10.1016/j.rcsop.2026.100724

**Published:** 2026-02-25

**Authors:** Mehdi Mohammadi, Artin Torshizi, Mohammadreza Heidari, Sholeh Ebrahimpour

**Affiliations:** a Department of Clinical Pharmacy, School of Pharmacy, Alborz University of Medical Sciences, Karaj, Iran; b Student Research Committee, Alborz University of Medical Sciences, Karaj, Iran

**Keywords:** Pharmacist, Patient simulation, Headache, Insomnia

## Abstract

This study investigated the practices of community pharmacists regarding two minor ailments, headache and insomnia, using a simulated patient (SP) method in Karaj, Iran. A trained pharmacy student, acting as an SP, visited pharmacies presenting with complaints of either headache or insomnia. Each simulation consisted of three steps: in Step 1, the SP explained the symptoms and waited for the pharmacist's recommendations; in Step 2, the SP requested medications; and in Step 3, the SP insisted on obtaining prescription-only drugs. A total of 200 pharmacies were surveyed; 100 were assigned to the headache scenario and 100 to the insomnia scenario. In the headache scenario, which required a referral to a physician, the pharmacists asked 26.4% of the patient history questions and addressed 9.4% of the physician referral criteria. Only 29 pharmacists (29.0%) referred the SP to a physician/imaging. Male pharmacists dispensed more prescription-only medications than female pharmacists (*p* = 0.03), whereas females were more likely to refer the SP to a physician (*p* = 0.02). In the insomnia scenario, which could be treated with nonprescription medications, the pharmacists asked 11.6% of the patient history questions and addressed 11.0% of the referral criteria. The most common recommendations in the first step were melatonin (52.0%), nonpharmacological interventions (18.0%), and herbal medicines (10.0%). Most pharmacists failed to obtain an adequate patient history, including physician referral criteria. In addition, the dosage, duration of therapy, and potential adverse drug reactions were not communicated to patients in most cases. The community pharmacists in this study demonstrated substantial deficiencies in history taking, contraindication screening, and patient counseling. These findings underscore the urgent need for targeted educational, regulatory, and practice-based interventions to enhance the quality and safety of over-the-counter care in Iran.

## 1 Introduction

Headache is a common neurological disorder. Globally, more than half of the adult population suffers from headache, making it the third leading cause of disability according to the Global Burden of Disease (GBD) report.^1^, ^2^ Between 1990 and 2017, headache was the most prevalent neurological disorder in Iran, accounting for over 99% of all reported neurological conditions and representing the leading cause of disability-adjusted life years (DALYs).^3^ In a face-to-face interview of more than 3600 Iranian individuals, over 76.0% reported at least one episode of headache within the past year.^4^ The use of over-the-counter (OTC) drugs to treat headache is widespread. However, concerns about medication-overuse headaches have risen due to the excessive and inappropriate use of OTC drugs.^5^ Inappropriate analgesic use is a key contributor to the development of chronic headache.^6^

Insomnia affects approximately 10% of the adult population worldwide, while an additional 20% experiencing occasional symptoms of sleep disorders.^7^ The prevalence of insomnia among Iranian adults is approximately 35%, highlighting it as a significant national health burden.^8^ Insomnia is often associated with physical and psychological problems^8^ and may signal a more serious underlying disorder.^9^ Data from the 2020 United States National Health and Wellness Survey indicated that greater insomnia severity is associated with poorer quality of life, reduced productivity, and increased healthcare resource utilization.^10^

Community pharmacists, as the most accessible healthcare professionals, play a crucial role in reducing self-medication, providing nonpharmacological management, dispensing OTC medications, and ensuring timely referral of patients to physicians. In Iran, practicing as a community pharmacist requires completion of a six-year academic program. In September 2019, the Iranian Ministry of Health released a national document emphasizing the importance of minor ailment management by community pharmacists. Despite this initiative, recent studies have reported significant gaps in Iranian pharmacists' knowledge and performance regarding OTC medications.^11^ Additionally, frequent dispensing of prescription-only medicines for minor ailments has been observed, potentially driven by workload pressures and limited time for thorough patient assessment.^12^

The use of simulation-based methodology dates back to the 1940s, when “mystery shopping” was employed in banks and retail stores to evaluate service quality in real-world settings. In 1973, Rosenhan applied this method in healthcare to assess the quality of care in a mental hospital by instructing simulated patients (SPs) to pretend to hear unreal voices.^13^ Since then, the use of the SP methodology to evaluate the quality of care in routine medical practice has steadily increased in healthcare settings.^14^ A recent systematic review indicated that patient simulation is a highly effective technique for evaluating pharmacist competence in pharmacy settings, particularly in terms of patient communication and consultation skills.^15^ Beyond evaluating healthcare services, simulated or “standardized” patients are valuable tools in medical education, and the current literature supports their effectiveness in enhancing student learning.^16^

This study examined the practices of Iranian community pharmacists in managing two minor ailments, headache and insomnia, via the SP method.

## 2 Methods

### 2.1 Study design, area, and setting

This cross-sectional study was conducted in community pharmacies in Karaj between December 2021 and May 2022.

### 2.2 Sample size

Assuming a 95% confidence level (Z = 1.96), a conservative expected proportion of appropriate practice of *p* = 0.50, and a margin of error of 3%, the initial sample size for an infinite population was calculated as 1068 via the formula *n = Z*^*2*^*p(1-p)/d*^*2*^. After correction for the finite number of community pharmacies in Karaj (*n* = 242),^17^ a final sample size of 198 pharmacies was needed. To compensate for potential unusable encounters, a 10% oversampling rate was applied, resulting in a final target sample size of 218 SP visits (pharmacies).

### 2.3 Simulated case scenarios

Two scenarios were developed: one for headache and one for insomnia. The headache scenario required medical referrals due to recent head trauma, whereas the insomnia scenario could be managed with OTC medications. For each scenario, a set of potential pharmacist questions and corresponding SP answers was determined in advance (Table 1).

**Table 1:** Study scenarios and request steps of the simulated patient.

Scenarios	Correct decision	Request steps		
		Step 1	Step 2	Step 3
Headache **Patient:** his brother; **Age**: 30 years; **Duration of symptoms**: about one week; **History of substance abuse, alcohol use, or smoking**: no; **Underlying disease**: none; **Drug history**: Pharmaton® (multivitamin-multimineral supplement) capsule once a day; **History of head injury**: 2 weeks ago, he fell while riding a bicycle, which caused a head injury; **Location of pain**: back of the head; **Fever**: no; **Blurred vision**: no; **Nausea and vomiting**: no; **Other symptoms**: no; **Intensity of pain**: moderate; **History of liver disease**: no; **Nature of headache**: nonthrobbing and uniform; **History of allergy**: no; **Factors that aggravate or relieve pain**: none; **Tried medications**: none.	Due to a history of severe head trauma within the last two weeks, the patient should have been referred to a physician for further evaluation.	Waiting for the pharmacist recommendations.	Ask for medications.	Ask for injectable analgesics, such as ketorolac, acetaminophen, etc.
Insomnia **Patient:** himself; **Age**: 24 years; **Duration of symptoms**: one week; **Manifestations**: difficulty in falling asleep and waking up; **History of drug abuse, alcohol, or smoking**: no; **Underlying disease**: none; **Drug history**: none; **Tiredness and boredom during the day**: yes; **Coffee or tea consumption**: no coffee, up to three cups of tea per day; **Measures to aggravate or relieve symptoms**: not tried.	Because there was no reason to refer the patient to a physician, he could be treated with a nonprescription medication such as antihistamines. In addition, sleep hygiene measures would be helpful.	Waiting for the pharmacist recommendations.	Ask for medications.	Ask for alprazolam or zolpidem.

Each scenario was simulated in three steps. In the first step, the SP presented with a complaint of headache or insomnia without providing additional details. If the pharmacist did not dispensed medication, recommended nonpharmacologic measures, or referred the patient to a physician, the simulation proceeded to the second step, during which the SP requested a medication. If the pharmacist dispensed OTC medicines, the SP proceeded to a third step, which was the request for prescription-only medications. These included injectable analgesics (acetaminophen or ketorolac) for headache and alprazolam or zolpidem for insomnia. If the pharmacist dispensed a prescription-only medication during the first or second step, the SP paid for it and then left the pharmacy. All medications obtained during the study were subsequently used for charitable purposes.

### 2.4 Study tools

For each scenario, a two-part checklist was developed based on nonprescription therapy reference books^18^, ^19^ to identify the key questions that the pharmacists were expected to ask. The first part of the checklist comprised patient history questions, including 11 items for the headache scenario and 9 items for the insomnia scenario. The second section addressed physician referral criteria that the pharmacists were expected to assess before determining the appropriate treatment. There were 11 criteria for headache and 3 for insomnia. In addition, all drug therapy consultations—including dosage, duration of use, administration, and adverse effects—along with any nonpharmacologic recommendations provided by the pharmacists, were documented. The SP completed the checklist for each pharmacist immediately after leaving the pharmacy. The appropriateness of pharmacologic and nonpharmacologic recommendations was evaluated based on the reference books mentioned above.

### 2.5 Training of the SP

A pharmacy student who volunteered to participate in the simulation-based study was trained by faculty members from the School of Pharmacy at Alborz University of Medical Sciences. Initially, a list of common minor ailments was compiled, and following discussion, headache and insomnia were selected for scenario development. Simulation scenarios were then developed with detailed patient information. Subsequently, during role-playing sessions, the SP and faculty members rehearsed each scenario multiple times, anticipating the possible questions that community pharmacists might ask. The study commenced once the faculty confirmed that the SP could consistently and accurately simulate the scenarios.

### 2.6 Data collection procedures

A comprehensive list of all pharmacies in Karaj, including their addresses, was obtained from the Food and Drug Administration. Pharmacies from all regions of the city were included. If the community pharmacist was absent or the pharmacy was closed during the SP visit, the pharmacy was excluded from the study, and the allocated scenario was conducted at the next pharmacy on the list. Each pharmacy was visited only once, with the two scenarios simulated alternately to ensure that only one scenario was conducted per pharmacy. To avoid receiving consultation from pharmacy technicians, the SP was instructed to confirm the identity of the pharmacist by checking their name tags.

### 2.7 Statistical analysis

Qualitative variables are presented as frequencies and percentages. The associations between pharmacist sex and the dispensing of prescription-only medications or physician referrals were investigated via the chi-square test. *P* values less than 0.05 were considered statistically significant. The data analysis was conducted via SPSS Statistics software (version 17.0).

## 3 Results

Of the 218 pharmacies approached, 16 had no pharmacist present, and 2 were closed, resulting in 200 of 242 pharmacies in Karaj being successfully visited. One hundred pharmacies were visited for the headache scenario, and another 100 were visited for the insomnia scenario. In each scenario, 55 pharmacists (55.0%) were male, and 45 (45.0%) were female.

Table 2 presents patient history questions and the frequency with which the pharmacists asked each question in each scenario. In general, only 26.4% and 11.6% of the patient history questions were asked in the headache and insomnia scenarios, respectively.

**Table 2:** Frequency of patient history questions asked by pharmacists (*N* = 200) during headache and insomnia scenarios.

Questions		Headache scenario n (%)	Insomnia scenario n (%)
Questions common to both scenarios	Age	31 (31.0%)	1 (1.0%)
	Duration of symptoms	64 (64.0%)	39 (39.0%)
	History of drug abuse, alcohol, or smoking	3 (3.0%)	12 (12.0%)
	Medications used for the current symptoms	23 (23.0%)	12 (12.0%)
	History of underlying diseases	44 (44.0%)	14 (14.0%)
	Past drug history	7 (7.0%)	12 (12.0%)
	Aggravating or alleviating factors	12 (12.0%)	2 (2.0%)
Questions specific to the headache scenario	Pain location	30 (30.0%)	–
	Accompanying symptoms	37 (37.0%)	–
	Nature of pain (sharp, dull, throbbing, etc.)	40 (40.0%)	–
	Allergy history	0 (0.0%)	–
Questions specific to the insomnia scenario	Daytime tiredness or feeling sleepy	–	3 (3.0%)
	Coffee or tea consumption	–	9 (9.0%)
Mean		26.4%	11.6%

The physician referral criteria for headache and insomnia and the frequency of pharmacists who asked each criterion are presented in Table 3. Notably, pharmacists assessed only 9.4% and 11.0% of the referral criteria for headache and insomnia, respectively.

**Table 3:** The frequency of asking about physician referral criteria by pharmacists (N = 200) in both study scenarios.

	Questions	Total n (%)
Physician referral criteria for headache	Severe headache	9 (9.0%)
	A headache intensified quickly.	0 (0.0%)
	Headache lasted more than ten days.	3 (3.0%)
	High fever or symptoms of infection	15 (15.0%)
	History of liver disease or consumption of more than three units (drinks) of alcohol per day	2 (2.0%)
	Neck stiffness	0 (0.0%)
	Any neurological changes (seizures, mood changes, visual disturbances, etc.)	8 (8.0%)
	High-risk patients (HIV, cancer, etc.)	0 (0.0%)
	Secondary headache (except sinus headaches)	29 (29.0%)
	Migraine-like symptoms (the migraine headaches have not yet been diagnosed)	31 (31.0%)
	Change in headache pattern	6 (6.0%)
	**Average percentage**	**9.4%**
Physician referral criteria for insomnia	Repeated midnight awakenings or early morning waking up	4 (4.0%)
	Chronicity (lasts more than three weeks)	2 (2.0%)
	Sleep problem that is secondary to another disorder	27 (27.0%)
	**Average percentage**	**11.0%**

### 3.1 Headache scenario

**Fig. 1:**
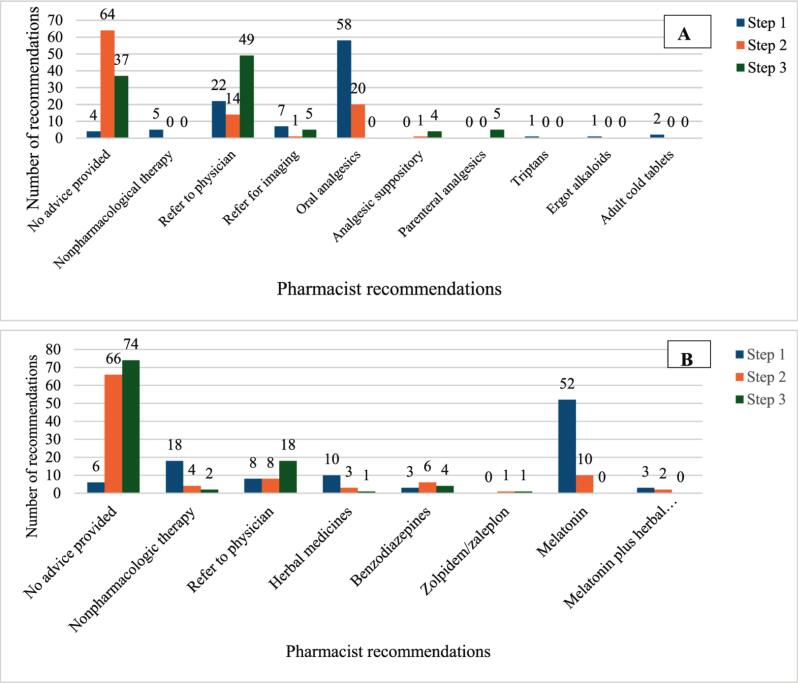
Frequency of pharmacist recommendations according to the simulation step for A: headache scenario B: insomnia scenario.

The pharmacist recommendations for the two scenarios according to the simulation steps are shown in Fig. 1. The most common recommendations in the first step of the headache scenario were oral analgesics (58.0%), referral to a physician (22.0%), and patient referral for imaging (7.0%). Five pharmacists recommended nonpharmacological therapy, and all were inappropriate measures. These included fluid intake in 3 patients and evaluation for visual disorders in 2 patients. A history of head trauma within the last 2 weeks was a physician referral criterion. Eighteen pharmacists (18.0%) asked about a history of recent head trauma, of whom 8 (8.0%) referred the patient to a physician and 7 (7.0%) referred him directly for brain imaging.

In the second step, when the simulated patient requested a medication, 64 pharmacists (64.0%) did not provide any advice, 20 (20.0%) dispensed oral analgesics, and 14 (14.0%) referred the patient to a physician. Finally, in the last step, when the simulated patient requested a parenteral analgesic, 49 pharmacists (49.0%) chose to refer the patient, 37 (37.0%) did not provide any advice, and 5 (5.0%) dispensed injectable analgesics.

Acetaminophen/ibuprofen/caffeine (57.6%), acetaminophen (10.9%), and nonsteroidal anti-inflammatory drugs (NSAIDs) (5.4%) were the most common medications recommended by the pharmacists. The medications recommended by the pharmacists for both scenarios are shown in Supplementary Table 1.

The associations between pharmacist sex and patient referrals to a physician were investigated. During all three steps of the simulation, 39 patients were referred to a physician by male pharmacists, and 46 patients were referred by female pharmacists, which was a statistically significant difference (*p* = 0.02). Parenteral analgesics were dispensed by 5 pharmacists, all males. None of the female pharmacists recommended parenteral analgesics (*p* = 0.03).

The pharmacists dispensed 87 nonparenteral medications in all three steps of the simulation. Counseling regarding dosage, duration of use, and adverse drug effects was performed for 24 (27.6%), 10 (11.5%), and 2 (2.3%) prescriptions, respectively.

### 3.2 Insomnia scenario

As shown in Fig. 1B, the most common pharmacist recommendations in the first step of the simulation were melatonin (52.0%), referral to a physician (18.0%), and herbal products (10.0%). Pharmacists did not provide any advice in most second- (66.0%) or third- (74.0%) step simulations.

Melatonin (62.5%), herbal products (14.6%), and alprazolam (8.3%) were the most common medications recommended by the pharmacists for insomnia (see Supplementary Table 1). None of the pharmacists recommended antihistamine use. The appropriate nonprescription medicines for insomnia and headache are shown in Supplementary Table 2.

Twenty-four pharmacists (24.0%) recommended nonpharmacological modalities to treat insomnia; 5 of them were incorrect, including using yogurt or yogurt drinks, reading books, avoiding sesame, and going out for a walk. Adherence to sleep hygiene practices was the most common nonpharmacological suggestion given by the pharmacists in this scenario.

Eleven male and 4 female pharmacists dispensed prescription-only medications in all steps of the simulation, and the difference was not statistically significant (*p* = 0.18). Moreover, physician referrals were performed by 18 male and 16 female pharmacists (*p* = 0.13).

In all steps, the pharmacists prescribed 96 medications. Counseling regarding dosage, duration of use, and adverse drug effects was performed for 55 (57.3%), 24 (25.0%), and 3 (3.1%) prescriptions, respectively.

## 4 Discussion

The main finding of this study was significant deficits in the history-taking skills of the pharmacists, including physician referral criteria. In addition, patient counseling regarding dispensed medications was not performed appropriately. Under the FIP–WHO *Good Pharmacy Practice* guidelines, pharmacists are required to maintain full awareness of patients' relevant medical histories, including diagnoses, laboratory data, and prescription or nonprescription medicines.^20^34.

Pharmacists are the most accessible healthcare professionals and provide effective patient education and consultation.^21^ Taking a detailed patient history to identify those who need referral to a physician and providing patient consultation regarding pharmacological and nonpharmacological therapies are among the most critical skills that pharmacists need to fulfill their vital role.^22^ In this study, the pharmacists asked only 26.4% and 11.6% of the patient history questions for the headache and insomnia scenarios, respectively. Our findings are similar to those of another patient simulation study for headache in three cities in Jordan.^23^ Hammad et al. evaluated the practices of 38 community pharmacists regarding the nonprescription management of headache and reported that only 9.9% of questions concerning patient history were asked. In an SP study by Bernhard et al. in Potsdam, Germany, 42 community pharmacies were approached for headache medication. The median counseling score was only 3 out of 9 points, revealing significant gaps in pharmacists' headache-related counseling.^24^ A comparable study from Brazil reported that no pharmacists recommended nonpharmacological strategies for headache management, and their counseling skills were rated as “poor to fair”.^25^ In a similar study, Soltani et al. evaluated the practices of Iranian pharmacists in patients with sore throat and dysuria.^12^ They reported that more than 90% of the pharmacist consultations lasted less than one minute, and in more than half of the cases, antibiotics were inappropriately recommended. The allocated time for providing OTC medications was less than 3 min in 85% of the cases in Gilson et al.'s study.^26^ Another SP study in Jordan investigated the practices of community pharmacists regarding insomnia management.^27^ In line with previous findings, the study revealed that the pharmacists did not inquire about patients' medical histories or other symptoms in any of the cases. It cannot be overemphasized that obtaining a detailed patient history is crucial for determining the need for referral to a physician and for providing appropriate patient consultations regarding medications and nonpharmacological interventions. Notably, we could not measure the duration of consultations in our study because of frequent interruptions of consultations during the study.

One of the key components of patient consultation in managing minor ailments is identifying criteria for physician referral. In this study, pharmacists assessed only 9.4% and 11.0% of the referral criteria for headache and insomnia, respectively. In the headache scenario, a history of head trauma (causing secondary headache), which was asked by only 18.0% of the pharmacists, was a physician referral criterion. However, most pharmacists (83.3%) who asked about head trauma referred the SP to a physician or brain imaging department for further evaluation.

In general, our findings revealed that history-taking was not convincing in either scenario. One possible solution might be the use of mobile health (mHealth) technology to improve pharmacists' performance, as evidenced by a study by Paydar et al. in Iran.^28^ In their study, an OTC therapy application was developed and evaluated to help pharmacists improve their knowledge and pharmaceutical skills in OTC therapy. Pharmacists managed a minor ailment once with their knowledge and once by using the application. In the headache scenario, the median (IQR) score of the pharmacists increased from 5.5 (2–8) to 21 (14–23.5), out of a total score of 28, after the application was used. The corresponding values for sleep disorders were 4 (1.5–7.5) and 15.5 (14.5–18.5) out of a total score of 24. For both disorders, the pharmacist scores after using the application were significantly higher than their own knowledge.

The commonly recommended OTC medications in this study were acetaminophen and NSAIDs (as monotherapies or in various combinations) for headache and melatonin and herbal products for insomnia. Although drug selection was appropriate, the patients were not properly counseled about dosage, duration of use, or common adverse reactions to medications. Interestingly, no pharmacist dispensed antihistamines, even though this class is recommended as a first-line nonprescription medication for insomnia.^18^

In the headache scenario, parenteral analgesics were dispensed for only 5 patients, all after patient insistence during step 3 of the simulation. Prescription-only medications for insomnia were prescribed to less than 10% of the patients during all simulation steps. This rate was substantially lower than that reported in a study in Pakistan, where benzodiazepines were dispensed to 61.8% of the 270 SPs who received medications for insomnia.^29^ This difference may be explained by the different methodologies used in the Pakistani study. In their study, only 13 (3.5%) SPs were managed by pharmacists, and the majority (83.8%) were handled by pharmacy technicians. Conversely, the SP in our study was first asked to recognize the pharmacist and then request medication. Therefore, the lower rate of benzodiazepines dispensing in our study could be attributed to the pharmacists' familiarity with nonprescription therapy. The patient simulation method was also used in Newcastle and Sydney, Australia, to evaluate the practices of pharmacies in terms of insomnia management.^30^ The results of their study revealed that medications were dispensed in 96% of the patients. Antihistamines and herbal medicines, with frequencies of 65% and 31%, respectively, were the most recommended medications. Nonpharmacological recommendations, such as avoiding stimulant drinks, activities before sleep, and relaxation strategies, were given to only 24% of the patients. The corresponding value in our study was approximately 20%, which highlighted the deficits in the practices of pharmacists with regard to insomnia.

Regardless of the dispensed medication, the pharmacists in this study did not appropriately counsel patients about the dose, duration, and common adverse effects of the drugs. Wazaify et al. reported similar results; the dose and time of administration were the only information provided by the pharmacists in less than half of the SPs complaining of insomnia.^27^ In addition, the pharmacists did not provide recommendations regarding drug interactions, contraindications, lifestyle changes, or sleep hygiene in the study. Our findings were also in line with the results of the Soltani et al. study, which revealed the suboptimal practices of pharmacists regarding efficient patient counseling and education.^12^

In this study, there were no differences between the practices of male and female pharmacists regarding history taking or the questioning of the referral criteria (data not shown). In the headache scenario, female pharmacists showed a greater tendency to refer the SP to a physician and a lower tendency to recommend parenteral analgesics. However, such a difference in practice was not observed in the insomnia scenario. This discrepancy has also been reported in other studies that examined gender differences in the practices of community pharmacists. In a cross-sectional study conducted in Poland, female pharmacists more frequently endorsed the need for physician–pharmacist collaboration and expressed stronger support for pharmacists' involvement in shared patient care.^31^ Such attitudes may plausibly translate into greater readiness to refer patients when clinical uncertainty is present. However, the Polish study assessed professional attitudes rather than actual referral behavior, and existing evidence on gender-related differences in pharmacists' clinical decision-making remains limited and context dependent. In contrast, Soltani and colleagues^12^ reported no significant difference between male and female pharmacists in the rate of antibiotic dispensing for the simulated patient presenting with symptoms of sore throat or dysuria. Therefore, although our findings are consistent with documented gender differences in collaborative orientation, they should be interpreted with caution. Additional research is warranted to determine whether the pattern observed in our setting persists across different populations, practice environments, and clinical scenarios.

In addition to history taking, the rational management of a patient with headache or insomnia requires a structured clinical approach that includes symptom assessment, the evaluation of warning signs, physician referrals, and nonpharmacologic recommendations.^32^ Our findings suggest that this broader clinical reasoning process is underutilized in practice, as reflected by limited assessment and frequent reliance on medication supply alone. Despite not being recorded in this study, communication skills also appeared to be suboptimal among pharmacists. Although the Ministry of Health document emphasized the expanding clinical role of pharmacists in minor ailment management, the present findings suggest that these competencies have not yet been consistently translated into routine practice. Notably, the Iranian Food and Drug Administration have an established surveillance system to monitor pharmacies regarding the provision of patient consultations, and all pharmacists are aware of this system. The observed deficit in patient counseling may be attributed to multiple factors, including gaps in pharmacy education and curriculum, limited opportunities for internships or practical training, the absence of standardized pharmacy practice guidelines, lack of remuneration for consultation services, high workload, and the prevailing public perception of pharmacists as primarily dispensers of medication rather than healthcare providers.^11^, ^33^ Targeted continuing professional development programs, practical clinical algorithms for OTC symptom management, and system-level incentives for patient counseling may help bridge this gap and increase the quality and safety of pharmacy-based care. Providing financial incentives seems to encourage pharmacists to provide appropriate patient services. A future study investigating the attitudes and practices of pharmacists in relation to financial incentives would be interesting.

The key strength of this study is that the SP method can reliably reflect real-world pharmacist practice. This method has been widely used in previous studies. A recent systematic review of SP studies in community pharmacies, primarily from the Middle East and North Africa, revealed widespread deficiencies in pharmacists' history taking, counseling, and referral practices.^34^ The review also highlighted substantial methodological variability across studies and emphasized the need for standardized SP-based evaluation frameworks.

### 4.1 Limitations

• The study was conducted in a single city, which may limit the generalizability of the findings.
• Only two disorders, headache and insomnia, were simulated in this study. Pharmacists may behave differently in other scenarios.

### 4.2 Recommendations

Practical implications for community pharmacists

• Targeted continuing professional education programs on structured history taking, patient assessment, warning sign recognition, and appropriate referrals should be implemented.
• The routine use of standard clinical algorithms or clinical decision-supporting applications should be promoted.
• Integration of nonpharmacological recommendations as a part of routine OTC counseling is recommended.

Policy and educational recommendations

• Appropriate monitoring systems and incentive structures for high-quality counseling and safe practices should be introduced.
• Reinforcing undergraduate and postgraduate training in clinical reasoning, differential diagnosis, risk assessment, and patient communication is recommended.
• The alignment between pharmacy curricula, national competency frameworks, and real-world practice requirements should be improved.

## 5 Conclusions

This investigation revealed significant deficits in history-taking skills among the included pharmacists and inadequate pharmacological and nonpharmacological recommendations for patients with headache and insomnia complaints. Although national policy emphasizes an expanded role for pharmacists in the management of minor ailments, our findings suggest that current educational practices require strengthening to adequately prepare community pharmacists for this role. Because the SP method is a reliable and internationally accepted approach for evaluating the quality of patient care in real-world settings, the findings of this study accurately reflect existing gaps in pharmacy practice in Iran. Although OTC counseling is a core component of the pharmacy curriculum in Iran, the absence of a fee-for-service model may partially explain the observed shortcomings.

## Clinical trial number

Not applicable.

## CRediT authorship contribution statement

**Mehdi Mohammadi:** Writing – review & editing, Methodology, Formal analysis, Conceptualization. **Artin Torshizi:** Writing – review & editing, Investigation, Data curation, Conceptualization. **Mohammadreza Heidari:** Writing – original draft, Validation, Formal analysis. **Sholeh Ebrahimpour:** Writing – review & editing, Project administration, Methodology, Conceptualization.

## Consent for publication

Not applicable.

## Ethics approval and consent to participate

The study was conducted in accordance with the Declaration of Helsinki. Ethical approval was obtained from the Research Ethics Committee of Alborz University of Medical Sciences (ID: IR.ABZUMS.REC.1400.269). Informed consent was obtained from the simulated patient. To assess the real-world performance of pharmacists, the ethics committee approved a waiver of informed consent. In accordance with ethical approval, the names of pharmacies and pharmacists were not recorded on the checklist. The study findings were subsequently reported to the Food and Drug Administration for further analysis.

## Funding

No funding was received for the study or publication of this research.

## Declaration of competing interest

The authors declare that they have no known competing financial interests or personal relationships that could have appeared to influence the work reported in this paper.

## Data Availability

The datasets used and/or analyzed during the current study are available from the corresponding author upon reasonable request.

## Appendix A Supplementary data

Supplementary material Image 1
