# Supplementary material for: Pharmacist-led responses to headache and insomnia symptoms: A simulated patient study
Source: Explor Res Clin Soc Pharm. 2026 Feb 25;22:100724. doi: 10.1016/j.rcsop.2026.100724 (PMC12969350; doi:10.1016/j.rcsop.2026.100724)
Supplement: Supplementary file 1 — Supplementary material [file mmc1.docx]

| Supplementary Table 1. Frequency of medications recommended by pharmacists according to request steps in the insomnia and headache scenarios | | | |
| --- | --- | --- | --- |
|  | Step 1 | Step 2 | Step 3 |
| **Insomnia Scenario** | | | |
| Melatonin | 52 | 10 | 0 |
| Melatonin/herbal | 3 | 2 | 0 |
| Herbal products | 10 | 3 | 1 |
| Chlordiazepoxide | 2 | 2 | 1 |
| Alprazolam | 1 | 4 | 3 |
| Zolpidem | 0 | 1 | 1 |
| **Headache scenario** | | | |
| Acetaminophen | 8 | 2 | 0 |
| Acetaminophen codeine | 4 | 0 | 0 |
| Acetaminophen/caffeine | 1 | 1 | 0 |
| Acetaminophen/caffeine/ibuprofen | 38 | 15 | 0 |
| Oral NSAIDs | 7 | 2 | 0 |
| NSAIDs suppositories (diclofenac, naproxen, indomethacin) | 0 | 1 | 4 |
| Parenteral NSAIDs | 0 | 0 | 5 |
| Triptans | 1 | 0 | 0 |
| Common cold products | 2 | 0 | 0 |
| Ergotamine/caffeine | 1 | 0 | 0 |

| Supplementary Table 2. Recommended non-prescription medicines for insomnia and headache | |
| --- | --- |
| **Insomnia** | **Headache** |
| First-generation antihistamines (diphenhydramine) | Acetaminophen |
| Melatonin (controversial) | Nonsteroidal anti-inflammatory drugs (ibuprofen, naproxen, ASA) |
| Valerian products (controversial) | Magnesium salicylate |
|  | Caffeine (in combination with acetaminophen or nonsteroidal anti-inflammatory drugs |
